# Clinical validation of a model predicting the risk of preterm delivery

**DOI:** 10.1371/journal.pone.0171801

**Published:** 2017-02-09

**Authors:** Yohann Dabi, Sophie Nedellec, Claire Bonneau, Blandine Trouchard, Roman Rouzier, Alexandra Benachi

**Affiliations:** 1 Service de Gynécologie-Obstétrique, AP-HP, Hôpital Antoine Béclère, Université Paris Sud, Clamart, France; 2 Cellule de Transfert in Utero - Ile de France, Hôpital Antoine Béclère, Clamart, France; 3 Service de Chirurgie, Institut Curie, Saint-Cloud, France; 4 EA 7285: Risques cliniques et sécurité en santé des femmes et en santé périnatale, Université, Versailles-St-Quentin-en-Yvelines, France; BC Children's Hospital, CANADA

## Abstract

**Objectives:**

To validate a model predicting the risk of threatened preterm delivery and to establish the optimal threshold for this risk scoring system.

**Materials and methods:**

Two cohorts were studied: one of singleton pregnancies without preterm premature rupture of membranes (PPROM) and no cervical cerclage (cohort 1) and one of twin pregnancies without PPROM and no cervical cerclage (cohort 2). Patients were included from January 1^st^ 2013 until December 31^st^ 2013 by the Regional Perinatal Network of Ile de France with patients transferred because of threatened preterm delivery at 22 to 32 weeks of gestation. The individual probability of delivery within 48 hours of admission was calculated using the nomogram for every patient. Discrimination and calibration of the nomogram as well as the optimal threshold were determined using R studio.

**Results:**

The nomogram accurately predicted obstetric outcome. Discrimination and calibration were excellent, with an area under the curve (AUC) of 0.88 (95% CI 0.86–0.90) for cohort 1 and 0.73 (95% CI 0.66–0.80) for cohort 2. The optimal threshold would be 15% for cohort 1 and 10% for cohort 2. Using these thresholds, the performance characteristics of the nomogram were: sensitivity 80% (cohort 1) and 69% (cohort 2), negative predictive value 94.8% (cohort 1) and 91.3% (cohort 2). Use of the nomogram would avoid 253 unnecessary transfers in cohort 1.

**Conclusions:**

The nomogram was efficient and clinically relevant in our high risk population. A threshold set at 15% would help minimize the risk of preterm deliveries in singleton pregnancies and should reduce unnecessary, costly and stressful in utero transfer.

## Introduction

Recent progress in the management of threatened preterm delivery has led to an increased survival rate of newborns through the development of new tocolytic drugs, the use of antenatal corticosteroids, and the organization of maternity wards by level of care according to gestational age. It is well known that newborn survival rate is higher when the delivery takes place in a maternity hospital with an appropriate level of care [1 –3]. As such, women with threatened preterm delivery may be transferred from one hospital to another. It is therefore important to determine correctly and efficiently who should be transferred. In fact, more than half of all women admitted for preterm labor are delivered after 37 weeks of gestation [4]. To assess the risk of preterm delivery, scoring systems combining clinical variables consistently associated with preterm delivery have been proposed. These systems have low predictive value and high false-positive rates and therefore are obsolete [5, 6]. To our knowledge, there is no reliable and validated tool that predicts whether a woman admitted for threatened preterm delivery is indeed going to give birth within 48 hours.

Nomograms are models designed to help clinical decision making when assessing patient risk and outcome [7, 8]. A nomogram to predict preterm delivery developed by Allouche et al. in 2011 has a high positive-predictive value [9], but has only been tested in one cohort and therefore is not widely used. Prospective validation of a nomogram in a clinical setting is essential to improve decision-making reliability, and so we tested the aforementioned nomogram. The main objective of our study was to validate the nomogram as a reliable tool for helping clinicians dealing with hard decisions to transfer or not patients presenting with high risk threatening preterm delivery. Our second main objective was to establish the optimal threshold for the model.

## Materials and methods

### Details of ethics approval

Our study was non interventional. Since 2013, every time a midwife from the Regional Perinatal Network of Ile de France was in charge with transferring a patient from an hospital to another, she calculated the risk of effective preterm delivery for the patient using the nomogram. At the time of the transfer, patients were aware the data collected might be used for medical and / or research purpose and gave their oral consent.

We sought for ethic consent from our Institutional Review Board in 2014 when we decided to analyze the data for the purpose of our study and realize an external validation of the nomogram. No written consent was required nor asked at that time as the study was non interventional and oral consent has been obtained earlier.

This study was approved by our Institutional Review Board on October 24, 2014.

Reference number: Comité d’Ethique de Recherche en Obstétrique et Gynécologie OBS 2014-09-06.

All clinical investigation has been conducted according to the principles expressed in the Declaration of Helsinki.

### Model parameters

The nomogram we tested [9] (available at http://www.perinatology.com/calculators/TRANSFER.htm) estimates two probabilities: 1. The probability of delivery within 48 hours, and 2. The probability of delivery before 32 weeks of gestation. As the threat of immediate delivery requires urgent transfer, we included in the analysis only the probability of delivery within 48 hours.

The following six parameters were used to calculate individual scores for the risk of delivery: number of fetuses, duration of pregnancy (weeks and days), cervical length (mm), vaginal bleeding, preterm premature rupture of membranes (PPROM), and uterine contractions requiring tocolysis.

To assign gestational age, we used first- or early second-trimester sonography. If these data were not available, gestational age was calculated using the first day of the last period. PPROM was diagnosed as leakage of amniotic fluid prior to initiation of labor. Leakage had to be identified objectively by direct examination, the use of nitrazine tests, or rapid identification of insulin-like growth factor-binding protein-1 in cervicovaginal secretions.

### Study population

Data on patients transferred to a level 3 maternity unit because of threatened preterm delivery from January 1^st^ 2013 to December 31^st^ 2013 were collected using the Ile de France Regional Perinatal Network. All relevant data to calculate the nomogram were collected prospectively, at the time of the transfer. All patients evaluated for transfer between 22 and 32 weeks of gestation upon admission were included, after exclusion of patients with PPROM and / or cervical cerclage. The clinician in charge with the patient was responsible with the decision to transfer his patient to another maternity and was in charge of contacting the Ile de France Regional Perinatal Network.

The Ile de France Regional Perinatal Network is an independent entity that acts as an intermediate between different maternity units. It main role, besides confirming the need for in utero transfer, is to find an appropriate maternity ward for admission of the patient.

Patients were divided into two groups. The first group included patients with a singleton pregnancy without PPROM and no cervical cerclage (cohort 1) and the second group included patients with a twin pregnancy without PPROM and no cervical cerclage (cohort 2).

### Definition of terms

In utero transfer was defined as transfer of pregnant women between hospitals using any means of transportation. The Ile de France Regional Perinatal Network was in charge of coordinating the transfer between maternity care centers.

We used the classification of French regulations on the safety of childbirth (dated Oct. 9, 1998) to determine the level of perinatal care. Level 1 centers have no neonatal units and can only accept newborns in optimal health. Level 2 centers have the required neonatal facilities to care for neonates born after 32 weeks of gestation. Level 3 centers have an intensive care unit for the weakest newborns and neonatologists are onsite 24/7.

All level 3 centers were using the same protocols for threatening preterm birth management according to National Guidelines edited by the National College of Obstetrics and Gynecology.

Threatened preterm delivery was diagnosed when uterine contractions were associated with clinical modifications of cervical length. Uterine contractions were reported by patients and monitored using tocography. Cervical length was clinically evaluated and measured sonographically, but only the sonographic measurement was used in the nomogram. Cervical length was measured when the entire cervical canal was visualized as the distance between the internal and the external orifice. All sonographic measurements were performed by experienced investigators at the time of admission.

Treatments of threatened preterm delivery were administered to every woman and were continued during the transfer. These included the use of betamethasone as antenatal corticosteroid therapy and tocolytic drugs (calcium channel blockers, β–mimetics or atosiban).

### Statistical analyses

#### Establishing ROC curves and calibrating the model

Individual risk scores were plotted to produce a receiver operating curve (ROC) and to calibrate the model. The performance of the model was quantified with respect to discrimination and calibration. Discrimination was quantified using the area under the ROC. By comparing agreement between predicted probability and observed pregnancy outcome, calibration was studied graphically using calibration curves. These curves represented grouped proportions vs mean predicted probability in groups defined by quartiles. Performance of the calibration was evaluated using the unreliability index U [10].

All analyses were performed using the R package with the rms library (http://lib.stat.cmu.edu/R/CRAN/). We used the curves and the “presence-absence” R package to determine the optimal threshold for the nomogram.

#### Defining optimal threshold

The optimal threshold could be defined as the probability given by our model above which patients should definitely be transferred since the risk of delivering within 48 hours is higher than the risk of a useless transfer with no delivery. An ideal threshold minimizes “false negatives” and so avoids non-transfer of patients who will deliver within 48 hours.

Several optimal thresholds could be chosen for the nomogram depending on the aim, including sensitivity, specificity, correctly classified patients, and kappa coefficient. The minimum ROC distance threshold was the most relevant for this study. It is defined as the minimum distance between the ROC curve and the upper left angle of the frame.

## Results

From January 1st 2013 to December 31st 2013, 736 patients were transferred from their original maternity care center to a level 3 maternity unit using the Ile de France Regional Perinatal Transfer Network for threatened preterm delivery. Data were received for all patients such that there was a zero dropout rate.

**Fig 1:**
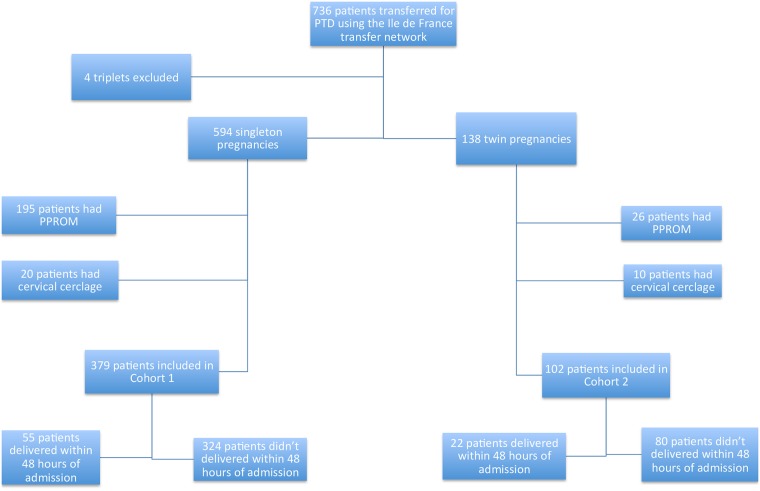
Study flow diagram of the 736 patients transferred using the Ile de France transfer network. Patients with PPROM and / or cervical cerclage were excluded as well as 4 triplet pregnancies. Final cohorts included 379 singleton pregnancies among which 55 patients delivered within 48 hours of admission and 102 twin pregnancies among which 22 delivered within 48 hours of admission.

Of the original sample (N = 736), 379 patients met the inclusion criteria for cohort 1 (singleton pregnancy without PPROM and no cervical cerclage) and 102 met the criteria for cohort 2 (twin pregnancy without PPROM and no cervical cerclage). In total, data from 481 patients were included in our statistical analyses (Fig 1).

Table 1 summarizes the individual and obstetric characteristics of the women included in our analyses. Around half of our patients were nulliparas and we had few grand multiparas (≥3 deliveries) Only 13.5% of patients in cohort 1 and 8.8% in cohort 2 had a history of preterm delivery or late miscarriage. Mean gestational age was 28 weeks and 2 days. Most patients had a functional cervical length ≤15 mm (53.1% of patients in cohort 1 and 48.4% in cohort 2). Data on cervical length were missing for 10.6% of patients in cohort 1 and 10.7% in cohort 2.

**Table 1:** Individual and obstetrical characteristics of the women included in the study (n = 481).

Patients	Cohort 1: Singleton pregnancies without PPROM or cervical cerclage, n = 379	Cohort 2: Twin pregnancies without PPROM or cervical cerclage, n = 102
**Age (y), mean**	30.1	32
≤ 18, n (%)	1 (0.3%)	0 (0%)
≥ 35, n (%)	76 (20.1%)	26 (25.5%)
**Nulliparas**, n (%)	213 (56.2%)	48 (47.1%)
**Multiparas** (≥3)	20 (5.2%)	5 (4.9%)
**History of preterm delivery or late miscarriage,** n (%)	51 (13.5%)	9 (8.8%)
**Gestational age at transfer** (weeks)		
Mean	28.2	28.3
≤ 24 wks, n (%)	9 (2.4%)	3 (2.9%)
25–28, n (%)	166 (43.8%)	37 (36.3%)
29–32, n (%)	204 (53.8%)	62 (60.8%)
**Clinical characteristics at admission**		
Vaginal bleeding, n (%)	37 (9.8%)	5 (4.9%)
Uterine contractions requiring tocolysis, n (%)	263 (69.4%)	77 (75.5%)
**Functional cervical length** (a) *		
Mean, mm	15.4	16.3
≤ 15	180 (53.1%)	44 (48.4%)
≤ 25 and > 15	121 (35.7%)	35 (38.5%)
> 25	38 (11.2%)	12 (13.2%)
**Obstetrical outcome: delivery within 48 h after transfer**, n (%)	55 (14.5%)	22 (21.6%)

*Data not available for:
Cohort 1 singleton pregnancies: 40 Cohort 2 twin pregnancies: 11

### Prediction of the probability of delivery within 48 hours after in utero transfer based on clinical and sonographic variables

**Fig 2:**
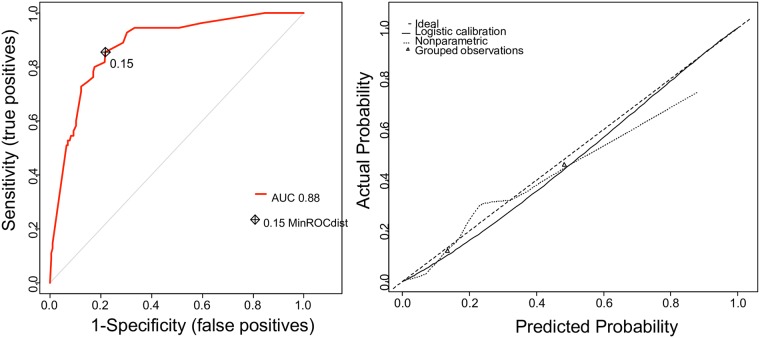
**On the left: Discrimination of the prediction model for delivery within 48 hours of admission for cohort 1.** ROC curve of the nomogram for the prediction of premature birth within 48 hours. Concordance index: 0.88 (95% CI: 0.86–0.90). **On the right: Calibration of the prediction model for delivery within 48 hours of admission for cohort 1.** The x-axis represents the probability of delivery within 48 hours after transfer calculated using the nomogram, and the y-axis represents the actual rate of delivery within 48 hours. The dashed line represents the performance of an ideal nomogram. The predicted and observed rates of delivery within 48 hours are plotted as the grouped observations and logistic calibration.

We used a prediction model for each cohort separately. In cohort 1, 55 patients (14.5%) delivered within 48 hours of admission. The model revealed an AUC of 0.88 (95% CI 0.86–0.90). Discrimination and calibration of the nomogram in predicting delivery within 48 hours for cohort 1 are reported in Fig 2.

The nomogram accurately predicted obstetric outcome. The predicted and observed rates of delivery within 48 hours were highly concordant, with no statistical difference (U: p < 0.05). These results demonstrate that the individual probability of delivering within 48 hours after transfer can be predicted by combining routinely available clinical information and sonographic measurement of uterine cervical length.

**Fig 3:**
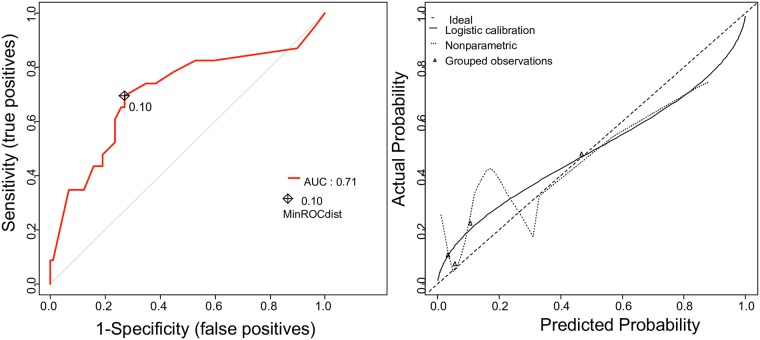
On the left: Accuracy of the prediction model for delivery within 48 hours of admission for cohort 2. ROC curve of the nomogram for the prediction of premature birth before 48 hours after admission. Concordance index: 0.73 (95% CI: 0.66–0.80). **On the right: Calibration of the prediction model for delivery within 48 hours of admission for cohort 2.** The x-axis represents the probability of delivery within 48 hours after admission calculated using the nomogram, and the y-axis represents the actual rate of delivery within 48 hours. The dashed line represents the performance of an ideal nomogram. The predicted and observed rates of delivery within 48 hours are plotted as the grouped observations and logistic calibration.

As for cohort 2, 22 patients (21.6%) delivered within 48 hours of admission. The model revealed an AUC of 0.73 (95% CI 0.66–0.80). Calibration accuracy was determined as in cohort 1. Calibration was good with no statistical difference between prediction and observation (p < 0.05). Discrimination and calibration of the nomogram in predicting the probability of delivery within 48 hours after admission for cohort 2 are reported in Fig 3.

Overall, the nomogram accurately predicted the individual probability of preterm delivery within 48 hours.

### Defining the optimal threshold clinically relevant for everyday use of the nomogram

The nomogram was clinically useful in decision making regarding transfer of patients with threatened preterm delivery

**Fig 4:**
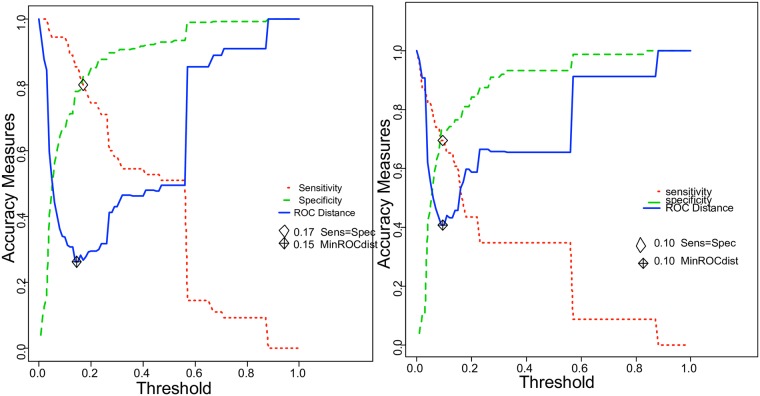
Risk of preterm birth within 48 hours: Optimal threshold determination. **On the left: In cohort 1**: To determine the best preterm risk threshold (minimization of false-negative and false-positive rates), one reliable statistical tool was used: MinROCdist. MinROCdist is the cut-off that minimizes the distance between the ROC curve and the upper left corner of the unit square. ***On the right*: *In cohort 2*:** To determine the best preterm risk threshold (minimization of false-negative and false-positive rates), one reliable statistical tool was used: MinROCdist. MinROCdist is the cut-off that minimizes the distance between the ROC curve and the upper left corner of the unit square.

For cohort 1, the minimum ROC distance revealed an optimal threshold of 15%. Using this threshold, we would have avoided 253 unnecessary transfers (patients who were transferred but did not deliver within 48 hours). Eight patients (2.1%) who should have been transferred would not have been (Fig 4).

The performance characteristics of the nomogram using this threshold were sensitivity 80%, specificity 82%, positive predictive value (PPV) 39.8% and negative predictive value (NPV) 94.8%.

For cohort 2, the minimum ROC distance revealed an optimal threshold of 10%. Using this threshold, we would have avoided 63 unnecessary transfers. Six patients (5.9%) who should have been transferred would not have been (Fig 4).

The performance characteristics of the nomogram using this threshold were sensitivity 69%, specificity 73%, PPV 48.5% and NPV 91.3%.

## Discussion

Preterm labor is a stressful condition for mothers and doctors, especially when it occurs in a small maternity unit ill—equipped for the management of premature births. Most patients transferred because of threatened preterm labor do not deliver following the transfer. Therefore a model allowing doctors to transfer patients at high risk when appropriate is needed to avoid stressful and costly unnecessary transfer.

Our external validation of the model published by Allouche et al. demonstrated its efficiency in predicting individual risk of preterm delivery within 48 hours of admission. To our knowledge, this is the first model with such a high predictive value and low false-negative rate. We also defined the optimal threshold to help clinical decision making regarding patient transfer. Women at high risk with a singleton pregnancy without PPROM and without cerclage, and with a predicted risk of preterm delivery over 15% should be transferred.

The objective of our study was not to discuss the parameters included in Allouche’s model and it was definitely not attempting to optimize the nomogram. Indeed, their methodology to build the nomogram was appropriate. Moreover, the parameters they included are consistent with the main prognosis factors we use to evaluate the severity of a threatening preterm birth in everyday practice. We wanted to test the nomogram on a large cohort to evaluate its efficiency as a clinical making tool to help the clinician.

Since our data were provided from a centralized transfer cell, we couldn’t retrieve information regarding patients that clinicians didn’t consider for transfer i.e. patients not at high risk for effective preterm delivery. However, this was not the point of this nomogram and it was definitely not the aim of its creation. Indeed, patients not considered for transfer to a higher maternity level were obviously not considered really threatening and practitioners considered that the probability they may deliver preterm was low. The population of patients we considered is the one praticians were worried about enough to ask for transfer in a maternity with appropriate level of care. This “high risk” population is the one the nomogram was built for and is the one we aimed to validate the nomogram on.

We included only patients without PPROM in our analyses. This was an a priori and not data-driven decision since we considered these patients to be too unstable and with a high risk of preterm delivery. We also excluded patients with cervical cerclage because they are also unstable: 6/20 patients with a singleton pregnancy, no PPROM and cervical cerclage delivered within 48 hours (30%). By excluding patients with PPROM and cervical cerclage, we meant cohorts 1 and 2 to be representative of patients that clinicians would deal with in everyday practice and for whom the decision to transfer should be discussed.

Even if no decision was taken based on the results of the nomogram, midwives of the Ile de France Regional Perinatal Network calculated for each patient included the individual probability of delivery within 48 hours. As so, there were no drop-outs during the year covered by the study meaning that delivery status 48 hours after admission was known for every patient transferred. This information is especially important since the nomogram must be easily manageable to be used in everyday practice and indeed, it was.

Some data were missing (Table 1), especially cervical length at inclusion, but the nomogram still allowed evaluation for every patient of the probability of delivery within 48 hours with missing data. Furthermore, not all maternity units are equipped to measure cervical length at admission, so our cohorts may be representative of the patients the nomogram might be useful for. We limited our inclusion to 22 gestational weeks at least since this seemed the earliest acceptable for transfer. Indeed, before 22 weeks, no center in France, would consider active management (including intensive neonatal care for the newborn) in case of delivery.

The nomogram was excellent in discriminating patients in cohort 1, but was less conclusive in cohort 2. The AUC remains adequate, but the model needs to be improved to make it more efficient. In cohort 2 (twin pregnancies), 22 patients (21.6%) delivered within 48 hours after admission. Multiple pregnancies are high-risk and should be managed in an appropriate maternity care center from the beginning. Future studies should improve the efficiency of the nomogram in twin pregnancies.

The nomogram predicts the individual probability of preterm delivery within 48 hours and before 32 weeks. We focused on the probability of preterm delivery within 48 hours. This time is needed to allow complete steroid treatment and therefore to reduce neonatal complications due to prematurity [11]. The clinical decision to transfer a patient thus depends mainly on the outcome within 48 hours. External validation of the nomogram was needed before decisions could be based on the nomogram results alone.

By utilizing the nomogram, 316 transfers could have been avoided. Defining the exact economic gain is difficult since there are, to our knowledge, no studies that have evaluated the exact cost of unnecessary transfers. However, the economic issue of unnecessary transfer is well known and concerns all developed countries. This may become a key issue over the years that we will eventually have to address. We believe this model is a first step toward a reduction of transfer costs. Moreover, transferring patients can prove cumbersome and even traumatic for families. Preterm delivery is source of psychological distress [12] and avoiding unnecessary transfers would avoid additional stress. Avoiding such transfers may be an additional psychological benefit for patients and their families. These benefits need to be balanced against the ethical risk of morbidity and mortality for patients mistakenly not transferred using the nomogram, which in our study included 2.1% of patients with a singleton pregnancy. The cost of preterm deliveries may be considerable, even more than the cost of unnecessary transfers [13–15].

Identifying high-risk patients for preterm delivery remains difficult, even with sonographic measurement of cervical length. A recent Austrian study by Mailath-Pokorny et al [16] evaluated previously published prediction models and how to improve them. In a cohort of 617 patients, between 2007 and 2012, two modified prediction models were tested for assessment of preterm delivery risk within 48 hours. The AUC was 0.77 (95% CI: 0.71–0.82), compared with our value of 0.88 (95% CI 0.86–0.90). There are various potential reasons for such a difference: 1) The inclusion criteria: We voluntarily excluded patients with PPROM and cervical cerclage for the reasons previously explained, but Mailath-Pokorny et al. did not. 2) The proportion of pregnant woman admitted before 24 weeks was significantly higher than in Mailath-Pokorny et al. in the cohort previously used to validate their prediction models. 3) The number of twin pregnancies is not specified. The models used by Mailath-Pokorny et al. include two new variables, C-reactive protein and fetal fibronectin, which seem to improve accuracy moderately. For the 48-hour model, the AUC was 0.8 (95% CI: 0.70–0.81).

Few maternity units, however, are equipped to assay fetal fibronectin rapidly in vaginal secretions. Moreover, C-reactive protein level must be correlated with the onset of symptoms since there is a delay in detecting an increase in level.

The nomogram published by Allouche et al. has the advantage of being simple and applicable in almost every maternity unit. The model that includes C-reactive protein and fetal fibronectin should be evaluated in a population with more strict inclusion criteria, ie, no cerclage and no PPROM since patients at high risk are those concerned with its purpose.

## Conclusion

In summary, our nomogram has proven efficient and clinically relevant. Since no other relevant trial is available [17], it can only be presumed that this risk scoring system will help reduce adverse outcomes associated with inappropriate maternity ward deliveries. Whether the nomogram should be used as a simple indicator of an effective high risk of preterm delivery or to prevent unnecessary transfers is open to discussion.

## References

[1] Phibbs C. S., Bronstein J.M., Buxton E., and Phibbs R.H. “The Effects of Patient Volume and Level of Care at the Hospital of Birth on Neonatal Mortality.” *JAMA* 276, no. 13 (10 2, 1996): 1054–59. 8847767

[2] Shlossman P. A., Manley J.S., Sciscione A.C., and Colmorgen G.H. “An Analysis of Neonatal Morbidity and Mortality in Maternal (in Utero) and Neonatal Transports at 24–34 Weeks’ Gestation.” *American Journal of Perinatology* 14, no. 8 (9 1997): 449–56. 9376004 10.1055/s-2007-994178

[3] Towers C. V., Bonebrake R., Padilla G., and Rumney P. “The Effect of Transport on the Rate of Severe Intraventricular Hemorrhage in Very Low Birth Weight Infants.” *Obstetrics and Gynecology* 95, no. 2 (2 2000): 291–95. 10674596 10.1016/s0029-7844(99)00528-1

[4] Parant O., Maillard F., Tsatsaris V., Delattre M., Subtil D., Goffinet F., and EVAPRIMA Group. “Management of Threatened Preterm Delivery in France: A National Practice Survey (the EVAPRIMA Study).” *BJOG*: *An International Journal of Obstetrics and Gynaecology* 115, no. 12 (11 2008): 1538–46. 19035990 10.1111/j.1471-0528.2008.01929.x

[5] Papiernik E., and Kaminski M.. “Multifactorial Study of the Risk of Prematurity at 32 Weeks of Gestation. I. A Study of the Frequency of 30 Predictive Characteristics.” *Journal of Perinatal Medicine* 2, no. 1 (1974): 30–36. 4468289 10.1515/jpme.1974.2.1.30

[6] Honest H., Bachmann L.M., Sundaram R., Gupta J. K., Kleijnen J., and Khan K. S. “The Accuracy of Risk Scores in Predicting Preterm Birth—a Systematic Review.” *Journal of Obstetrics and Gynaecology*: *The Journal of the Institute of Obstetrics and Gynaecology* 24, no. 4 (6 2004): 343–59. 15203570 10.1080/01443610410001685439

[7] Eastham J.A., Kattan M.W., Scardino P.T. “Nomograms as Predictive Models.” *Seminars in Urologic Oncology* 20, no. 2 (5 2002): 108–15. 12012296 10.1053/suro.2002.32936

[8] Kattan M.W., Giri D., Panageas K.S., Hummer A., Cranor M., Van Zee K.J., et al “A Tool for Predicting Breast Carcinoma Mortality in Women Who Do Not Receive Adjuvant Therapy.” *Cancer* 101, no. 11 (12 1, 2004): 2509–15. 15495180 10.1002/cncr.20635

[9] Allouche M., Huissoud C., Guyard-Boileau B., Rouzier R., and Parant O.. “Development and Validation of Nomograms for Predicting Preterm Delivery,” Am J Obstet Gynecol 2011;204:242.e1–8 (Nomogram available at http://www.perinatology.com/calculators/TRANSFER.htm) 21093847 10.1016/j.ajog.2010.09.030

[10] Harrell F.E., Lee K.L., Mark D.B. “Multivariable prognostic models: Issues in developing models, evaluating assumptions and adequacy, and measuring and reducing errors.” (1996): Stat in Med 15:361–387. 8668867 10.1002/(SICI)1097-0258(19960229)15:4<361::AID-SIM168>3.0.CO;2-4

[11] “Effect of Corticosteroids for Fetal Maturation on Perinatal Outcomes. NIH Consensus Development Panel on the Effect of Corticosteroids for Fetal Maturation on Perinatal Outcomes.” *JAMA* 273, no. 5 (2 1, 1995): 413–18. 7823388 10.1001/jama.1995.03520290065031

[12] Singer L.T., Salvator A., Guo S., Collin M., Lilien L., and Baley J.. “Maternal Psychological Distress and Parenting Stress after the Birth of a Very Low-Birth-Weight Infant.” *JAMA* 281, no. 9 (3 3, 1999): 799–805. 10071000 10.1001/jama.281.9.799 PMC10189739

[13] Petrou S., Henderson J., Bracewell M., Hockley C., Wolke D., Marlow N., and EPICure Study Group. “Pushing the Boundaries of Viability: The Economic Impact of Extreme Preterm Birth.” *Early Human Development* 82, no. 2 (2 2006): 77–84. 16466865 10.1016/j.earlhumdev.2006.01.002

[14] Gilbert W. M. “The Cost of Preterm Birth: The Low Cost versus High Value of Tocolysis.” *BJOG*: *An International Journal of Obstetrics and Gynaecology* 113 Suppl 3 (12 2006): 4–9. 17206959 10.1111/j.1471-0528.2006.01117.x

[15] Petrou S., Mehta Z., Hockley C., Paula Cook-Mozaffari P., Henderson J., and Goldacre M. “The Impact of Preterm Birth on Hospital Inpatient Admissions and Costs during the First 5 Years of Life.” *Pediatrics* 112, no. 6 Pt 1 (12 2003): 1290–97. 14654599 10.1542/peds.112.6.1290

[16] Mailath-Pokorny M., Polterauer S., Kohl M., Kueronyai V., Worda K., Heinze G., et al “Individualized Assessment of Preterm Birth Risk Using Two Modified Prediction Models.” *European Journal of Obstetrics*, *Gynecology*, *and Reproductive Biology* 186C (1 8, 2015): 42–48. 10.1016/j.ejogrb.2014.12.010 25616254

[17] Davey M.A., Watson L., Rayner J.A, Rowlands S.. Risk scoring systems for predicting preterm birth with the aim of reducing associated adverse outcomes. *Cochrane Database of Systematic Reviews* 2011, Issue 11. Art. No.: CD004902. 10.1002/14651858.CD004902.pub4 22071815

